# Whole-genome sequences of *Helicobacter pylori* isolated from patients with high risk of gastric cancer in the Andes of Nariño, Colombia

**DOI:** 10.1128/mra.01232-23

**Published:** 2024-07-22

**Authors:** Kevin Andres Guzman, Angi Paola Daza, Rudy Viviana Gomez, Lidia Madeline Montenegro, Alvaro Pazos

**Affiliations:** 1 Grupo de investigación en Salud Pública, Centro de Estudios en Salud Universidad de Nariño (CESUN), Pasto, Colombia; 2 Departamento de Biología, Facultad de Ciencias Exactas y Naturales, Universidad de Nariño, Pasto, Colombia; Loyola University Chicago, Chicago, Illinois, USA

**Keywords:** *Helicobacter pylori*, genomes, gastric cancer, Colombia

## Abstract

We sequenced the complete genomes of 16 *Helicobacter pylori* isolates obtained from patients residing in Nariño, Colombia. These isolates were collected from patients presenting various gastric lesions classified according to the Correa cascade classification. The genomic characterization of these isolates provides valuable insights into the structure, composition, and pathogenicity.

## ANNOUNCEMENT

*Helicobacter pylori*, a Gram-negative microorganism, colonizes the gastric mucosa (1). This infection is recognized as the primary cause of inflammation, leading to diverse gastric lesions, including peptic ulcers and gastric cancer (2). In the Andean region of Colombia’s Nariño, the infection prevalence is around 90%, marking it as the area with the highest incidence rate (150 per 100,000) in the country (3).

The genome report comprises *H. pylori* isolates collected from 2017 to 2022 in Pasto City, Nariño, Colombia (1°12´36’’N 77°16’29’’W). The Nariño University Bioethics Committee (act number 58 of 10 April 2017) approved this study. All the participants signed the informed consent. Participants, with a mean age of 52 years (range: 30 to 68 years), included 3 men and 13 women. Histopathology results revealed that 11 patients had nonatrophic gastritis, while five exhibited intestinal metaplasia (Table 1)

**TABLE 1:** Summary of genome sequences reported^*a*^

Strain name	Sex	Age	Diagnosis	GenBank accession no.	No. of raw reads	SRA accession no.	Size genome	Genome coverage (×)	N50	GC%	N° contigs	Annotated genes	Phylogenomic analysis
AP002	F	52	IM	JAUQTH000000000	435,480	SRR26926616	1,668,722	78	31.913	39.22	157	1,649	hspColombia
AP015	M	50	IM	JAUQTI000000000	798,872	SRR26926615	1,689,940	142	47.025	39.23	185	1,692	hspColombia
AP018	F	47	IM	JAUQTJ000000000	901,960	SRR26926608	1,700,565	159	36.365	39.21	143	1,678	hspColombia
AP021	F	50	IM	JAUQTK000000000	861,644	SRR26926607	1,680,516	154	63.185	39.22	167	1,674	hspColombia
AP022	F	60	NAG	JAUQTL000000000	392,298	SRR26926606	1,638,615	72	107.077	38.86	52	1,595	hpEurope
AP025	F	65	CAG	JAUQTM000000000	278,462	SRR26926605	1,652,684	51	60.512	38.95	66	1,609	hpEurope
AP028	F	54	CAG	JAUQTN000000000	369,690	SRR26926604	1,673,711	66	38.220	39.20	167	1,685	hpEurope
AP031	F	52	CAG	JAUQTO000000000	301,156	SRR26926603	1,653,928	55	10.422	39.50	46	1,586	hspColombia
CR004	M	68	NAG	JAUQTP000000000	940,540	SRR26926602	1,680,991	168	87.648	38.95	90	1,627	hspColombia
CR005	F	54	NAG	JAUQTQ000000000	728,350	SRR26926601	1,649,918	132	73.150	39.09	80	1,613	hspColombia
CR031	F	50	NAG	JAUQTR000000000	732,372	SRR26926614	1,653,356	133	50.390	38.92	58	1,601	hspWAfrica
CR045	M	34	NAG	JAUQTS000000000	97,318	SRR26926613	1,687,749	18	63.377	39.07	127	1,593	hspColombia
CR047	F	62	NAG	JAUQTT000000000	401,636	SRR26926612	1,686,534	72	69.457	39.52	75	1,644	hspColombia
CR048	F	48	NAG	JAUQTU000000000	430,092	SRR26926611	1,692,916	76	58.084	38.85	73	1,649	hspColombia
CR054	F	30	NAG	JAUQTV000000000	731,098	SRR26926610	1,595,752	137	65.233	38.90	74	1,561	hspColombia
AP029	F	48	IM	JAUQTW000000000	247,298	SRR26926609	1,617,151	46	48.931	39.24	310	1,796	hspColombia

^*a*^ F: female; M: masculine; IM**:** intestinal metaplasia; NAG**:** non-atrophic gastritis; CAG: chronic active gastritis.

The gastric biopsy sample was cultured on Columbia agar (Oxoid, UK) supplemented with 10% sheep defibrinated blood, selective supplement Dent (Oxoid, UK), and 1% enrichment supplement Isovitalex (Oxoid, UK), under 10% CO_2_ at 37°C for 7 to 10 days. DNA extraction utilized the QIAmp DNA Mini Kit. Whole-genome sequencing was performed on the MiSeq TM platform (Illumina, Germany) using the Nextera XT kit (Illumina) for library preparation, resulting in paired ends of 2 × 300 bp and approximately 40× coverage. Sequencing raw data underwent quality control with FastQC v 0.12 software (4). Data integrity and precision were ensured through data cleaning using Trim Galore v 6.6 and Cutadapt v 4.3 software (5, 6). The novo genome assembly employed the SPAdes v 3.15.5 program (7). Genomes were annotated using the Prokaryotic Genome Annotation Pipeline (PGAP) v.6.5 in NCBI (8). Default parameters were used for all software tools.

A Multi-locus Sequence Typing (MLST) based on seven housekeeping genes of *H. pylori* (*atpA*, *efp*, *trpC*, *ppa*, *mutY*, *yphC*, and *urel*) was conducted using strains of PubMLST (https://pubmlst.org/). Aligned sequences were analyzed using Structure v 2.3.4 software (9). Phylogenomic analysis utilized MEGA v11 (10) on the core genome obtained with BIGSdb v.3 (11) of PubMLST using J99 reference strain (NZ_CP011330.1) with 1,484 reference genes. The tree was visualized and edited using the iTol v.6.8.1 (12).

**Fig 1:**
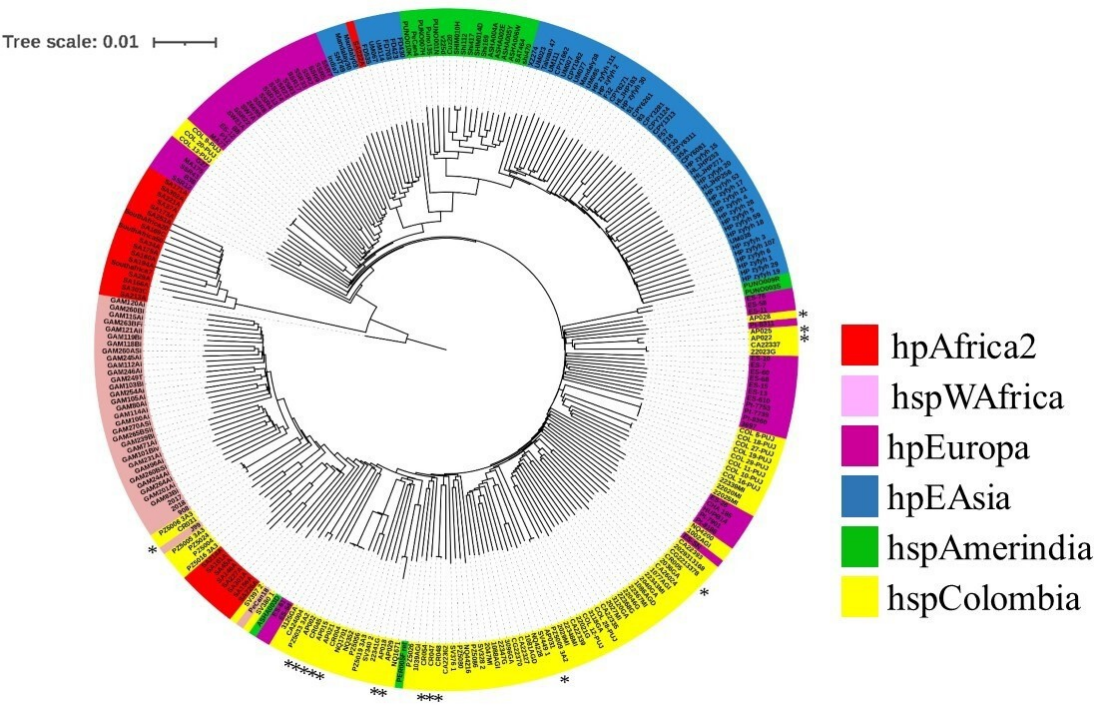
Phylogenomic tree of *H. pylori* isolates. The tree was constructed using 29 hpAfrica2, 35hspAmerindian, and 75 hspColombia reference isolates; the asterisk (*) denotes the 16 isolates under study. Each *H. pylori* isolation is color-coded with corresponding identifiers (IDs) displayed along the outer perimeter of tree. The legend provides details on the different populations (hp) and subpopulations (hsp) of *H. pylori*.

The phylogenomic analysis revealed 12 isolates forming a distinct lineage-specific to Colombia (hspColombia), alongside three samples of European origin (hpEurope) and one isolate from the African continent (hspWAfrica; Fig. 1). The structure analysis aligns with the phylogenomic findings, indicating that European isolates (hpEurope) exhibit predominantly European ancestry (>96%). Similarly, the isolate whose genomic component clusters with African strains displays a substantial percentage of African ancestry (92%; Table 1).

The hspColombia isolates exhibit a predominantly European and African ancestry, with a minimal proportion of Asian ancestry (hspAmerindian), suggesting that the lineage’s origin is a result of genomic mosaicism resulting from the intermixing of multiple strains following European colonization in the American continent.

Our findings offer an approximation of personalized medicine to allow the identification of ancestry and human-*H. pylori* coevolution that also allows an effective treatment to eradicate the infection (3, 13).

## Data Availability

This whole-genome shotgun project has been archived in the NCBI database under the accession number PRJNA984677. Detailed accession numbers for individual genomes are presented in Table 1.
